# Comparison of Safety and Immunogenicity of PVRV and PCECV Immunized in Patients with WHO Category II Animal Exposure: A Study Based on Different Age Groups

**DOI:** 10.1371/journal.pntd.0003412

**Published:** 2014-12-18

**Authors:** Yuan Fang, Li Chen, Man-Qing Liu, Zheng-Gang Zhu, Ze-Rong Zhu, Quan Hu

**Affiliations:** 1 Department of Neurology, Union Hospital, Tongji Medical College, Huazhong University of Science and Technology, Wuhan, China; 2 Jianghan District Centers for Disease Control and Prevention, Wuhan, China; 3 Wuhan Centers for Disease Control and Prevention, Wuhan, China; The Global Alliance for Rabies Control, United States of America

## Abstract

**Background:**

The aim of this study was to compare the safety and immunogenicity between purified vero cell rabies vaccine (PVRV) and purified chick embryo cell vaccine (PCECV) in patients with WHO category II animal exposure, especially in different age groups.

**Methodology/Principal Findings:**

In one-year clinical observation after vaccination with PVRV or PCECV under Zagreb (2-1-1) or Essen (1-1-1-1-1) regimens, information collection for the demographic and adverse events (AEs) and rabies virus laboratory examination of neutralizing antibody (RVNA) titers were performed for all patients with WHO category II animal exposure in Wuhan city. The results showed no significant differences of safety and immunogenicity between PVRV and PCECV both in Zagreb and Essen regimens. However, when compared with other age groups, most systemic AEs (36/61) occurred in <5-year-old patients, and <5-year-old patients have significant lower RVNA titer and seroconversion rate (RVNA ≥0.5 IU/ml) at day 7 both in Zagreb and Essen regimens or PVRV and PCECV groups.

**Conclusions:**

Our data showed that vaccination with PVRV is as safe and immunogenic as PCECV in patients of all age groups, but might be more popular for clinical use. When performing a vaccination with rabies vaccine in young children, the most optimal vaccine regimen should be selected.

## Author Summary

Nowadays, many approved vaccines with different components (such as purified vero cell rabies vaccine [PVRV], purified chick embryo cell vaccine [PCECV], and Human diploid cell vaccine [HDCV]) and many regimens with different vaccination schedules (Zagreb, Essen) are being used in the world. Thus, we compared the safety and immunogenicity between purified vero cell rabies vaccine (PVRV) and purified chick embryo cell vaccine (PCECV) in patients with WHO category II animal exposure, especially in different age groups. Our data showed no significant differences of safety and immunogenicity between PVRV and PCECV with Zagreb or Essen regimen in four age groups. However, compared with the other three age groups, young children aged less than 5 years have more systemic adverse events (AEs), and lower rabies virus neutralizing antibody (RVNA) titer and seroconversion rate (RVNA ≥0.5 IU/ml) at day 7 post-immunization. These findings highlight that it is important for young children, a population with more than 50% of human rabies deaths, to find the most optimal vaccine and vaccination schedule in the future.

## Introduction

Rabies, caused by rabies virus infection, remains a global health threat, and became the leading cause of infectious disease mortality in May 2006 in China [1]. In the world, Rabies is estimated to cause more than 55000 deaths every year, and is considered to be endemic in more than 150 countries and territories [2], [3]. Nowadays, China is in the midst of its third epidemic that begun in 1996 and peaked in 2007 (3300 cases), Wuhan, the largest city in the middle of China with about 10 million residents, has a medium incidence of rabies [4]. Although deadly, rabies can be prevented by timely initiation of post-exposure prophylaxis (PEP) which includes proper local treatment of bite wounds, administration of rabies vaccines either by intramuscular (IM) or intradermal (ID) route and local infiltration of rabies immunoglobulins (RIG) [5]. Due to high number of animal bites, there is a huge demand for rabies vaccines in developing countries of Asia and Africa [6].

Nowadays, purified chick embryo cell vaccine (PCECV) and purified vero cell rabies vaccine (PVRV) are currently recommended by WHO for PEP, and are being widely used in many countries in the world. In addition, compared to chick embryo cell, vero cell is a more practical manufacturing platform for vaccine production, which should be considered as an advantage of PVRV over PCECV. From 2001, PVRV has been successfully manufactured in China. ChengDa rabies vaccine (PVRV) was licensed by the Health Ministry of China and the State Food and Drug Administration of China (SFDA) in 2002 and has been marketed throughout the country since that time [7]. Although ChengDa PVRV under 2-1-1 regimen has been proved to be equally safe and immunogenic as the PCECV for PEP vaccination in adult volunteer [7], and has been marketed for more than 10 countries in the world, however, to our knowledge, there has been little reported about the safety and immunogenicity of PVRV or PCECV in different age groups, especially for young children. Thus we performed this study to compare the safety and immunogenicity of PVRV and PCECV under Zagreb and Essen regimens, especially in different age group patients with WHO category II animal exposure.

## Methods

**Figure 1:**
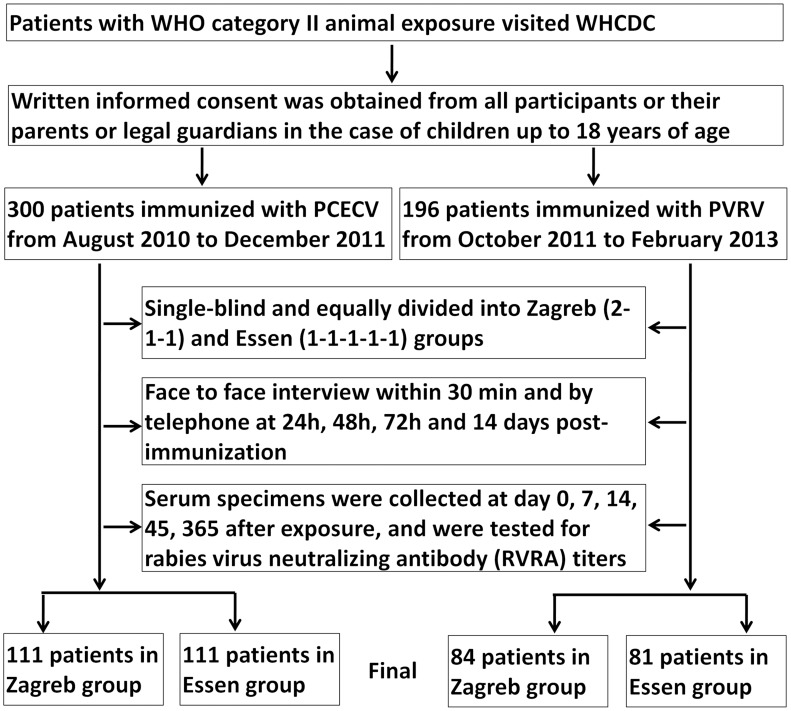
Flowchart of the safety and immunogenicity study.

From August 2010 to February 2013, the patients who visited the clinic of Wuhan Centers for Disease Prevention and Control (WHCDC), and were professionally evaluated as WHO category II exposure to suspected rabid animals according to WHO criteria for animal exposure (Nibbling of uncovered skin, minor scratches or abrasions without bleeding), were enrolled, and were divided single-blind and equally into two groups (Zagreb 2-1-1 and Essen 1-1-1-1-1) (Fig. 1). All patients lived in Wuhan for more than 6 months, and visited the clinic within 24 hours after exposure. The patients, who had chronic infectious diseases, or known hypersensitivity to any vaccine component, or received of rabies vaccine previously, were excluded. The protocol of this study was approved by the Institutional Review Board of WHCDC, and written informed consent was obtained from all participants, or their legal guardians in the case of children up to 18 years of age.

The sample size estimation was conducted according to the “Practical Manual of Sample Size Determination in Health studies” as described previously [8], a minimal of 75 cases in each group was required. The detailed study flow was shown in Fig. 1. For the patients in Zagreb and Essen groups, immunization with PVRV (Liaoning ChengDa Co., Ltd., Shenyang, China, 7.0 IU/0.5 ml/dose) or the imported PCECV (Rabipur, Novartis Vaccines and Diagnostics, 6.4 IU/1.0 ml/dose) was performed at day 0, 7, 21 or day 0, 3, 7, 14, 28 respectively. Safety monitoring was conducted by face-to-face observation after each immunization or by telephone during the study. In order to analyze the efficacy of vaccination, rabies virus neutralizing antibody (RVNA) titers in the serum were measured using a rapid fluorescent focus inhibition test (RFFIT) as described by Yu et al. [9]. Briefly, a constant dose of previously titrated, cell culture adapted, challenge virus (CVS-11) is incubated with serial dilution (three-fold serial dilution, from 1/3 to 1/6561) of the sera to be titrated. A reference serum (NIBSC, UK. The 2nd International Standard for Anti-Rabies) of known titer was included in each test. After one hour of incubation at 37°C, BSR cells (clone BHK21) were added into each well. After 24 h incubation, the estimation of the percentage of infected cells for each dilution of the sera allows determination of the titer of the unknown sera by comparing with the reference serum. Meanwhile, one of reference sera that we bought was sent to Chinese Center for Disease Control and Prevention for testing the antibody level to avoid deviation. Our data showed good reliability with assay variation of <15%. RVNA titers in sera were expressed as International Units per millilitre (IU/ml). Serum with titers ≥0.5 IU/ml, the WHO recommended protective level, was considered as a protective titer.

GraphPad Instat statistical software (GraphPad Software) was used for statistical analysis, and a P value of <0.05 was considered statistically significant. Where appropriate, data were expressed as mean ± standard deviation (SD) if not defined. Categorical variables were tested with chi-square of the Fisher exact test, and comparison between two groups was tested with the Student *t* test.

## Results

### Subjects

**Figure 2:**
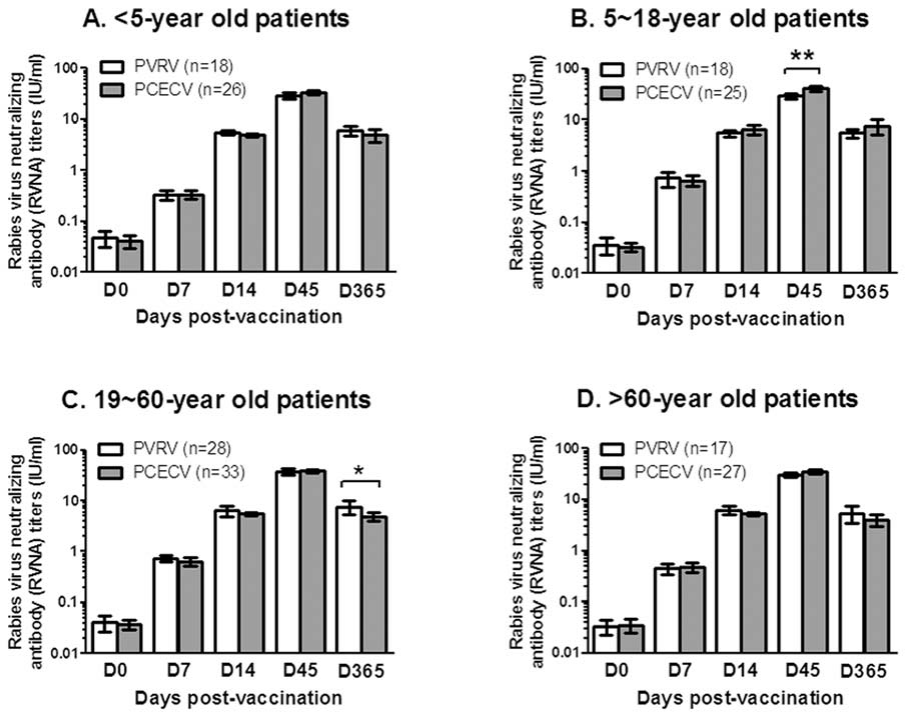
Comparison of RVNA titers between purified vero cell rabies vaccine (PVRV) and purified chick embryo cell vaccine (PCECV) vaccinated under Essen (1-1-1-1-1) regimen, the patients with WHO category II animal exposure were aged <5 years (A), 5–18 years (B), 19–60 years (C), and>60 years (D). Significant differences between two groups were found for 6-18-year old patients at day 45 (D45) (**p<0.01) and 19-60-year old patients at day 365 post-vaccination (*p<0.05). The data shown are the mean±95% confidence interval. Student *t* test was used for the statistical analysis.

**Figure 3:**
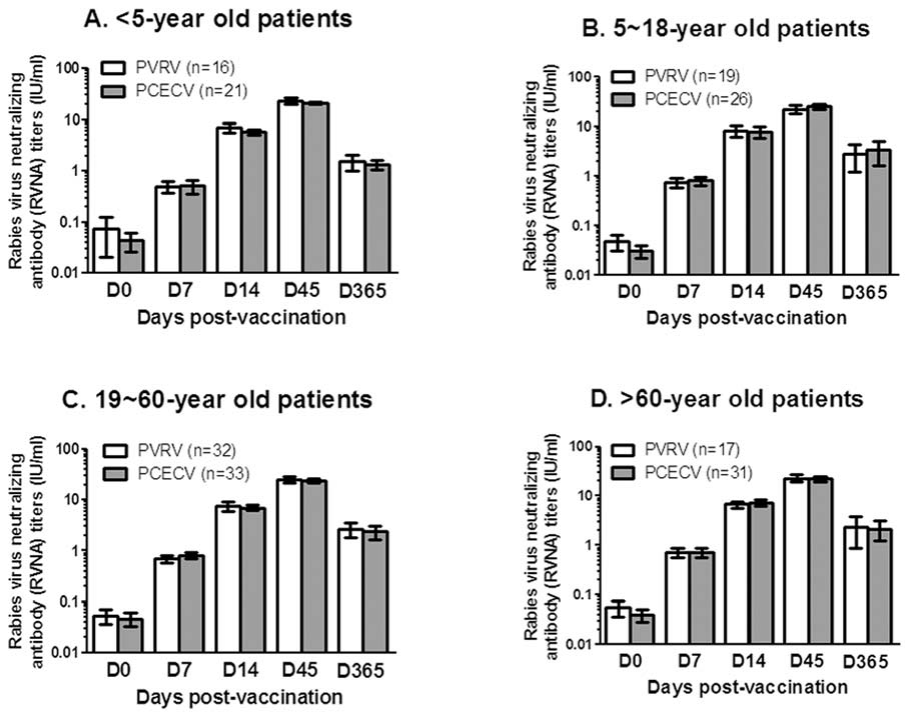
Comparison of RVNA titers between purified vero cell rabies vaccine (PVRV) and purified chick embryo cell vaccine (PCECV) vaccinated under Zagreb (2-1-1) regimen, the patients with WHO category II animal exposure were aged <5 years (A), 5–18 years (B), 19–60 years (C), and>60 years (D). No significant differences were found between two groups before (D0) or day 7 (D7), day 14 (D14), day 45 (D45), day 365 (D365) after rabies vaccination. The data shown are the mean±95% confidence interval. Student *t* test was used for the statistical analysis.

During the study period, 496 patients with WHO category II animal exposure were enrolled in this study. Finally, 387 patients have completed data sheet and blood collection, and a complete study flow was showed in Fig. 1. There are no significant differences between PVRV and PCECV groups on mean ages (p = 0.103 or 0.432 for Zagreb and Essen respectively), sex, and RVNA titers before immunization (Day 0 in Fig. 2 and Fig. 3). During the study period no patient was injected with RIG according to WHO post-exposure prophylaxis (PEP) measures for WHO category II animal exposure, and no patient developed clinical rabies.

### Safety analysis

In order to evaluate the safety of PVRV and PCECV in different age groups, both local adverse events (AEs) and systemic AEs were recorded during the study process. Table 1 showed the most common AEs in four age groups, of which no significant difference was found in the patients with AEs between PVRV and PCECV, even compared in different age groups or different administration regimens (Zagreb or Essen). However, most systemic AEs (36/61) occurred in <5-year old patients, and when analyzing the number of patients with the severity of fever (defined according to the “Preventive vaccine clinical trials, adverse events grading guidelines” issued by the China Food and Drug Administration), PCECV seemed to have more patients with medium fever (37.6∼39.0°C) than PVRV (P = 0.039, Table 1) for Zagreb, but no significant difference for Essen (P = 0.494, Table 1).

**Table 1:** Safety comparison between PVRV and PCECV with Zagreb or Essen regimen on different age group.

				Age groups for PVRV					Age groups for PCECV					P
				<5	5∼18	19∼60	>60	Total	<5	5∼18	19∼60	>60	Total	
**Zagreb**	Patients Number			16	19	32	17	84	21	26	33	31	111	0.539^d^
	Local AEs^a^			4	9	3	7	23	4	4	4	12	24	0.352^c^
		Pain		2	8	2	6	18	1	3	3	9	16	
		Induration		2	1	0	1	4	2	0	0	0	2	
		Edema		0	0	1	0	1	0	0	0	1	1	
		Tenderness		0	0	0	0	0	1	1	1	1	4	
	Systemic AEs^b^			8	2	2	2	14	12	4	2	3	21	0.685^c^
		Fever (°C)		7	1	1	1	10	9	1	1	1	12	0.039
			37.1∼37.5	5	1	0	0	6	1	0	0	0	1	
			37.6∼39.0	2	0	0	1	3	7	1	1	1	10	
			>39.0	0	0	1	0	1	1	0	0	0	1	
		Malaise		0	1	1	0	2	0	1	1	2	4	
		Allergy		0	0	0	0	0	1	1	0	0	2	
**Essen**	Patients Number			18	18	28	17	81	26	25	33	27	111	0.900^d^
	Local AEs^a^			3	7	2	4	16	4	10	3	8	25	0.644^c^
		Pain		1	5	0	2	8	0	7	2	5	14	
		Induration		1	1	0	1	3	2	1	0	1	4	
		Edema		0	0	1	1	2	1	1	1	1	4	
		Tenderness		1	1	1	0	3	0	1	0	1	2	
	Systemic AEs^b^			6	2	1	2	11	10	2	1	2	15	0.989^c^
		Fever (°C)		5	2	1	1	9	6	1	1	1	9	0.494^c^
			37.1∼37.5	4	1	0	0	5	2	1	0	0	3	
			37.6∼39.0	1	1	1	1	4	4	1	1	1	7	
			>39.0	0	0	0	0	0	0	0	0	0	0	
		Malaise		0	0	0	1	1	0	1	0	1	2	
		Allergy		0	0	0	0	0	2	0	0	0	2	

a Local adverse events (AEs) included pain, induration, edema, tendemess, and erythema in this study, but only top 4 AEs were listed.

b Systemic AEs included fever, malaise, allergy, restlessness, nausea and vomiting in this study, but only top 3 AEs were listed.

c By χ^2^ test for comparison of total patients with AEs between PVRV and PCECV.

d By χ^2^ test for comparison of patients number in different age groups between PVRV and PCECV.

### Immunogenicity analysis

The same to our previous data [8], in this study all patients have low RVNA titers of <0.5 IU/ml when enrolled, and reach a highest RVNA titers at day 45 for all vaccination methods (PVRV and PCECV) and regimens (Zagreb and Essen) (Fig. 2 and Fig. 3), of which all patients developed a protective RVNA titers of ≥0.5 IU/ml at day 14 and day 45. However, based on the data of RVNA titers on day 7, <5-year old patients seem to have significant lower seroconversion rates compared to other three age groups, especially for the patients with PCECV vaccination, and PVRV vaccination in Essen regimen (p<0.001, Table 2). In contrast, <5-year old patients immunized with PVRV have no significant difference (p = 0.114, Table 2) to>5-year old patients immunized with PVRV, but did have a similar low seroconversion rate compared to PCECV administered under Zagreb regimen (p = 0.957, Table 2). In addition, RVNA titers in patients aged>60 years also showed a significant difference to that of children and adults (aged 5∼60 years) only when injected with PVRV under Essen regimens at day 7 (p<0.05). When compared RVNA titers between PVRV and PCECV, both Zagreb and Essen groups have no significant differences in different age groups, only for 5-18-year old patients at day 45 and 19-59-year old patients at day 365 under Essen regimen (Fig. 2 and 3).

**Table 2:** Seroconversion rate (rabies virus neutralizing antibody (RVNA) titers ≥ 0.5 IU/ml) comparison between PVRV and PCECV with Zagreb or Essen regimen on different age groups at day 7 or day 365 post-immunization.

	Age groups	Day 7			Day 365		
		PVRV	PCECV	P^b^	PVRV	PCECV	P^b^
Zagreb	<5	43.8% (7/16)	42.9% (9/21)	0.957	93.8% (15/16)	90.5% (19/21)	0.715
	5∼18	73.7% (14/19)	80.8% (21/26)	0.572	94.7% (18/19)	96.2% (25/26)	0.821
	19∼60	75% (24/32)	90.9% (30/33)	0.053	93.8% (30/32)	90.9% (30/33)	0.666
	>60	76.5% (13/17)	77.4% (24/31)	0.940	88.2% (15/17)	78.8% (26/31)	0.678
	Total	69.0% (58/84)	75.7% (84/111)	0.303	92.9% (78/84)	90.1% (100/111)	0.494
	P^a^	0.114	**<0.001**		0.872	0.486	
Essen	<5	16.7% (3/18)	15.4% (4/26)	0.909	100% (18/18)	96.2% (25/26)	0.400
	5∼18	83.3% (15/18)	72% (18/25)	0.379	100% (18/18)	96% (24/25)	0.391
	19∼60	82.1% (23/28)	78.8% (26/33)	0.742	100% (28/28)	97.0% (32/33)	0.353
	>60	35.3% (6/17)	44.4% (12/27)	0.548	88.2% (15/17)	92.6% (25/27)	0.624
	Total	58.0% (47/81)	54.1% (60/111)	0.584	97.5% (79/81)	95.5% (106/111)	0.457
	P^a^	**<0.001**	**<0.001**		0.052	0.865	

a By χ^2^ test for comparison among four age groups.

b By χ^2^ test for comparison between PVRV and PCECV.

## Discussion

Nowadays, many approved vaccines with different components (such as PVRV, PCECV, and Human diploid cell vaccine (HDCV) [2]) and many regimens with different vaccination schedules (Zagreb, Essen [7], [8]) are being used in the world. However, which kinds of vaccines or regimens is the best choice for different age group patients remains unclear. In current study, we compared the safety and immunogenicity of PVRV and PCECV, especially in different age groups. Although only small number of patients was analyzed, our results indicated that PVRV had no significant difference of both safety and immunogenicity to PCECV, even in young children or elderly. However, because of availability, PVRV may be preferred by patients in developing country [7].

For the safety analysis, pain and fever were the most common AEs in local and systemic AEs respectively (Table 1), which was in agreement to our previous study [8], but different to the report by Madhusudana et al. [10]. The possible reason for the difference might be induced by different regimen, or patients with different exposure grade. In this study, PVRV showed non-inferiority to PCECV on safety both in Zagreb and Essen regimens, even in different age groups. However, although no difference of local AEs was found among different age group patients, most systemic AEs (59.0%, 36/61), especially for fever (67.5%, 27/40), occurred in young children (<5 years) (Table 1). When compared the severity of fever, more patients immunized with PCECV under Zagreb regimen had medium fever (37.6∼39.0°C) than the patients with PVRV (p = 0.039), which may be associated with the different volumes of vaccines (2 ml and 1ml at first time immunization with PCECV and PVRV, respectively), because the difference was not significant (p = 0.494, Table 1) when vaccinating with Essen regimen, a program with only one dose at first time immunization.

As pointed out by Gozdas et al. [11], it is not enough only to evaluate the safety of rabies vaccine after vaccination. Thus, we also analyzed the immunogenic profile over a one-year period. The mean RVNA titers based on different age groups and different immunization regimens were compared between PVRV and PCECV. No significant differences in RVNA titers between PVRV and PCECV in most of the age groups, neither Essen nor Zagreb regimen, were observed (Fig. 2 and Fig. 3). These results are in agreement with previous studies in India [10]. Interestingly, when comparing the seroconversion rate and RVNA titers of different age groups at day 7, the patients aged <5 years have significantly lower seroconversion rate (Table 2) and significantly lower antibody titers (Table 3) than that of ≥5-year old patients, which may be caused by the immature immune system of young children. Further, at day 7 the patients of <5-year immunized with Zagreb showed higher seroconversion rate than the patients of <5-year immunized with Essen regimen (43.8% vs 16.7% and 42.9% vs 15.4% for PVRV and PCECV respectively, Table 2), and no difference of seroconversion rate (p = 0.114) was observed within the Zagreb immunized group between <5-year versus ≥5-year patient groups immunized with PVRV under Zagreb (Table 2). Thus, because of no significant difference on the safety between Zagreb and Essen regimen, vaccination under Zagreb regimen in <5-year patients might be preferred.

**Table 3:** P values calculated with student *t* test for the comparison of the rabies neutralization antibody (RVNA) titers between <5-year old patients and other age groups at different time point or with different vaccination methods.

<5		Age groups under Essen regimen (1-1-1-1-1)			Age groups under Zagreb regimen (2-1-1)		
		5∼18	19∼60	>60	5∼18	19∼60	>60
Day 0	PVRV	0.2736	0.5310	0.1436	0.3243	0.3540	0.5214
	PCECV	0.2107	0.5448	0.3961	0.1393	0.7886	0.7068
Day 7	PVRV	**0.0019**	**<0.0001**	0.0913	**0.0085**	**0.0397**	**0.0243**
	PCECV	**0.0001**	**<0.0001**	**0.0341**	**0.0071**	**0.003**	**0.0486**
Day 14	PVRV	0.7899	0.3570	0.2012	0.3035	0.6431	0.7534
	PCECV	**0.0223**	**0.0226**	0.3465	0.0595	**0.0319**	**0.0369**
Day 45	PVRV	0.9652	**0.0350**	0.5690	0.8245	0.4500	0.8972
	PCECV	**0.0264**	0.0800	0.6409	**0.033**	0.1012	0.4397
Day 365	PVRV	0.5576	0.3387	0.5268	0.1394	0.0793	0.2816
	PCECV	**0.0491**	0.8452	0.2411	**0.0333**	**0.0335**	0.1663

In conclusion, our data showed that, under either Zagreb or Essen regimen, PVRV is equally safe and immunogenic as PCECV immunized in all age groups, and Zagreb regimen might be more suitable for young children to develop protective antibody as soon as possible. However, only small number of children, a population with more than 50% of human rabies deaths [12], was analyzed in this study, more data on the safety and immunogenicity for choosing a suitable vaccine and vaccination schedule for young children will be needed in the future.

## Supporting Information

S1 STROBE Checklist STROBE checklist for this study.
(DOC)
